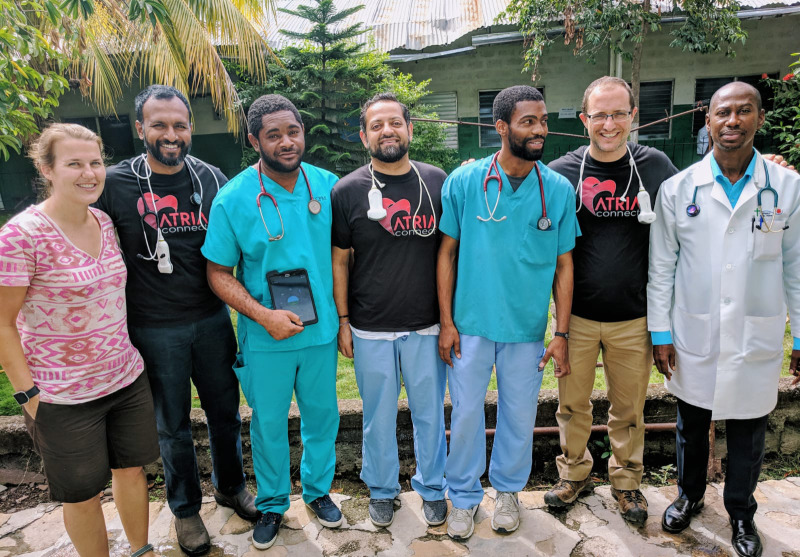# Demonstration of a Longitudinal Medical Education Model (LMEM) Model to Teach Point-of-Care Ultrasound in Resource-Limited Settings

**DOI:** 10.24908/pocus.v5i1.14226

**Published:** 2020-07-16

**Authors:** Michael Yao, Lauren Uhr, George Daghlian, Junedh M Amrute, Ramya Deshpande, Benji Mathews, Sanjay A Patel, Ricardo Henri, Gigi Liu, Kreegan Reierson, Gordon Johnson

**Affiliations:** 1 Division of Engineering and Applied Science, California Institute of Technology Pasadena, CA USA; 2 Department of Medicine, David Geffen School of Medicine, University of California Los Angeles, CA USA; 3 Division of Biology and Biological Engineering, California Institute of Technology Pasadena, CA USA; 4 Washington University School of Medicine St. Louis, MO USA; 5 Department of Hospital Medicine, HealthPartners Bloomington, MN USA; 6 Graduate Medical Education, OhioHealth Riverside Methodist Hospital Columbus, OH USA; 7 Alma Mater Hospital Haiti; 8 School of Medicine, Université Quisqueya Haiti; 9 Department of Medicine, Johns Hopkins University School of Medicine Baltimore, MD USA; 10 University of Minnesota Medical School Minneapolis, MN USA; 11 Legacy Emanuel Medical Center, Department of Internal Medicine, Legacy Health Portland, OR USA

**Keywords:** POCUS, Resource-limited, Longitudinal, Haiti, POCUS Training

## Abstract

**Background:** Short-term medical missions prevail as the most common form of international medical volunteerism, but they are ill-suited for medical education and training local providers in resource-limited settings. **Objective: **The purpose of this study is to evaluate the effectiveness of a longitudinal educational program in training clinicians how to perform point-of-care ultrasound (POCUS) in resource-limited clinics. **Design:** A retrospective study of a four-month POCUS training program was conducted with clinicians from a rural hospital in Haiti. The model included one-on-one, in-person POCUS teaching sessions by volunteer instructors from the United States and Europe. The Haitian trainees were assessed at the start of the program and at its conclusion by a direct objective structured clinical examination (OSCE), administered by the visiting instructors, with similar pre- and post- program ultrasound competency assessments. **Results:** Post-intervention, a significant improvement in POCUS competency was observed across six different fundamental areas of ultrasound (p < 0.0001). According to our objective structured clinical examination (OSCE), the mean assessment score increased from 0.47 to 1.68 out of a maximum score of 2 points, and each trainee showed significant overall improvement in POCUS competency independent of the initial competency pre-training (p < 0.005). There was a statistically significant improvement in POCUS application for five of the six medically relevant assessment categories tested. **Conclusion:** Our results provide a proof-of-concept for the longitudinal education-centered healthcare delivery framework in a resource-limited setting. Our longitudinal model provides local healthcare providers the skills to detect and diagnose significant pathologies, thereby reducing avoidable morbidity and mortality at little or no addition cost or risk to the patient. Furthermore, training local physicians obviates the need for frequent volunteering trips, saving costs in healthcare training and delivery.

## Background

Point-of-care medicine is a rapidly emerging field in diagnostic imaging that allows clinicians to administer acute care at the bedside in emergent situations 1. Namely, point-of-care ultrasound (POCUS) is a reliable diagnostic tool that affords clinicians the ability to examine patients in real time and make clinical assessments without the expense, delay, or radiation associated with other imaging modalities 2. Given its profound versatility, usability, and low training requirements, POCUS is emerging as an increasingly important tool for bedside diagnosis 3, 4, 5, 6, 7, 8, 9, 10, 11, 12.

With reduced costs and a user-friendly portable interface, POCUS holds promise for application in resource-limited settings that often suffer from a lack of access to diagnostic imaging technologies 13. Previous studies have shown POCUS to be effective in developing countries. For instance, Shah et al. showed that in two district rural hospitals the utilization of ultrasound changed the course of patient care up to 43% of the time 14, while Kotlyar et al. reported a ultrasound-driven change in patient management in 62% of cases in a tertiary care center in Liberia 15. Furthermore, POCUS education was shown to improve patient care by Mathews et. al., who developed a standardized protocol of POCUS education that resulted in long-term retention of ultrasound image acquisition and interpretation skills 16.

Given the well-documented benefits of POCUS, applying this technology to resource-limited areas would be opportune. Traditionally, international volunteering trips to resource-limited settings are centered around ‘short-term medical missions’ (STMMs), which are largely considered to be cost-ineffective and limited in timespan 17. One primary driver of this low efficacy is that many pathologies, especially in resource-limited areas, cannot be detected and/or treated in the duration of a single medical mission. Most medical interventions in these settings often require delivery of delayed lab results or treatment of complications secondary to initial treatment, which precludes clinical follow-up especially in the timeframes of typical STMMs 17. As a result, the existing medical infrastructure is left to bear the consequences of treating an acute influx of patients with already limited resources. Given these drawbacks, follow-up post-STMM treatment may potentially adversely affect the existing patient population as a result of a diversion and dilution of limited medical resources 17. Specifically, the use of POCUS in medical volunteering trips requires additional training that many clinicians practicing in low-resource medical institutions lack, making incorporating POCUS into medical practice challenging.

In this study, we propose a novel educational model, ‘longitudinal medical education model’ (LMEMs), and discuss how this model transcends many of the limitations faced by traditional STMMs. The LMEM model provides a unique framework for sustained medical education while minimizing costs and burden on the instructors. This was accomplished through an extended program that emphasizes hands-on training at the bedside, as opposed to the traditional patient care-oriented model of STMMs. Traditional short courses can get trainees familiar with POCUS, but their skills can often decay overtime as a result of a lack of longitudinal repetition. Alternatively, trainees are more likely to retain skills and continue utilizing POCUS as a part of their standard practice with a longitudinal training program over months, rather than an intensive, traditional two- or four- day course. However, many volunteer POCUS experts are unable to dedicate many months away from their respective practices to teach such longitudinal courses. 

To address this limitation, we created multiple teams of two to three instructors where each team visits the low-resource medical clinic for one week, spread out over a four-month period. This provided continuous mentorship for the POCUS trainees over the course of many months and allows for longitudinal hands-on training and evaluation. Furthermore, utilizing an array of instructors from Europe and the United States provides a broad perspective in teaching POCUS. The objective of the bedside training sessions is to work with the trainees individually and teach them fundamental POCUS skills through real-time clinical scenarios. Training local physicians reduces the need for frequent volunteering trips in the long run and has the potential to save tremendous costs in healthcare delivery. We show that by equipping and training Haitian physicians with state-of-the art diagnostic tools, such as POCUS, we can correctly diagnose many prevalent pathologies that often go misdiagnosed due to a lack of training and resources.

## Methods

We retrospectively evaluated the efficacy of the proposed LMEM model implemented over the span of four months. Study participants were clinicians from the 68-bed Alma Mater Hospital in Gros-Morne, Haiti, a small rural community on the north side of the Caribbean island. The program consisted of 12 local Haitian physicians, with 4 of the 12 completing the full 4-month training program while the remaining 8 engaged in a fraction of the training program. Our POCUS educators were practicing physicians across a wide variety of specialties including radiology, internal medicine, family practice, and emergency medicine.

### Program Description

The goal of our hospital-based POCUS training program was to demonstrate a proof-of-concept methodology in incorporating the usage of POCUS in resource-limited settings to ultimately improve the accuracy and timeliness of diagnoses and safety of procedures by utilizing ultrasound guidance. Our POCUS training program consisted of nine weeks of in-person training sessions spread over the course of four months. There were two week-long sessions for a general introduction to ultrasound, followed by seven weeks of POCUS training with one- to two- week separation periods interspersed throughout the training program. The overall training program consisted of a total of six ultrasound fundamentals: neck, lung, abdomen, cardiac, lower extremity, and soft tissue (Table 1). The curriculum was tailored to the individual specialties of the volunteer POCUS instructors on each week-long trip; collectively, they were able to cover a variety of hands-on concepts, as detailed in Table 1. Our pre- and post-training program objective structured clinical examination (OSCE) assessments were designed to assess the basics of POCUS, and were adapted from the Comprehensive Hospitalist Assessment and Mentorship with Portfolios (CHAMP) ultrasound program from Mathews et al 16. All volunteer instructors followed a standard set of ultrasound education principles to guide the in-person training of participants.

**Table 1:** Fundamentals of the ultrasound assessment

**Examination:**	**Fundamentals of Identifying and Interpreting POCUS Images:**
Neck ultrasonography	Carotid artery in transverse and longitudinal planes Color Doppler of carotid artery Internal jugular vein in transverse and longitudinal planes
Lung ultrasonography	Pleural line (lung sliding and A lines) Presence of pleural effusion Costophrenic recess
Abdominal ultrasonography	Liver and hepatorenal space Spleen and splenorenal space Kidneys and bladder in transverse and longitudinal planes Abdominal aorta in transverse and longitudinal planes
Echocardiography	LV, RV, LA, aorta, mitral and aortic valves, and pericardium in parasternal long axis Papillary muscles in parasternal short axis Four chambers and valves in the apical four-chamber view Hepatic vein and IVC in longitudinal axis with IVC entry into RA in subcostal view
Lower extremity ultrasonography	Femoral vein and artery with compression Greater saphenous and popliteal vein with compression
Musculoskeletal soft tissue ultrasonography	Achilles tendon Skin Muscle

### Online Modules and Supplemental Reading

Online training modules were provided by SonoSim, Inc. and featured a handheld probe simulator for interactive feedback. We asked the study participants to review the relevant online modules prior to the weekly group sessions that focused on a particular POCUS topic. For example, the participants were asked to review the echocardiography online module from SonoSim, Inc. prior to a week-long, in-person didactic sessions with the echocardiography specialist.

This online module-based training was supplemented with Point-of-Care Ultrasound POCUS-training textbook from Soni et al, which also acted as a reference guide for the study participants 18.

### Bedside POCUS Training

As aforementioned, the core of our POCUS training program involved a series of nine week-long, hands-on training sessions that together formed a single LMEM. The program utilized the Philips Lumify portable ultrasound system consisting of their S4-1 cardiac, C5-2 curvilinear, and L12-4 linear transducers. The Lumify system remained on-site indefinitely for continued usage by the trainees after the conclusion of the training program.

### Reacts-enabled Tele-ultrasound Training and Collaboration

In addition to the in-person training provided by volunteer POCUS experts, we used the Lumify with Reacts (Remote Education, Augmented Communication, Training and Supervision) integrated tele-ultrasound software developed in collaboration by Philips ultrasound and Innovative Imaging Technologies (IIT), Inc. in order to work with the study participants remotely in real-time. Reacts is a collaborative platform developed by IIT that allows for real-time remote medical education and consultation. The hand-held ultrasound system simultaneously streams ultrasound images along with video and audio in real-time. The system was used to continue ultrasound education during the limited number of weeks where POCUS educators were not available to travel to Haiti. These remote training sessions were conducted approximately three times over the course of the four-month data collection period using a reliable cellular or Wi-Fi connection.

### Assessments

Trainees were assessed in six core areas of ultrasound in terms of imaging and interpretation: neck, lung, abdomen, cardiac, lower extremity, and soft tissue. In each of these areas of assessment, there were multiple skills, and participants were evaluated on a scale of 0 to 2 for each skill. A score of 0 represents the inability to generate the ultrasound image, 1 represents the ability to generate the ultrasound image but an inaccurate interpretation of the image, and 2 represents the ability to both generate and interpret the ultrasound image. This ultrasound skills assessment was adapted from the CHAMP ultrasound program 16.

### Statistical Analysis

Assessment scores per core area with multiple skills were reported as the average of values, with the minimum possible score being 0 and the maximum being 2. Comparisons of assessment scores were performed using paired-samples t-test. Differences were considered statistically significant when p-value was less than 0.05. Statistical Package for the Social Sciences (SPSS) (IBM SPSS Statistics for Mac, Version 26.0. Armonk, NY: IBM Corp) was used for analysis. 

## Results

**Figure 1:**
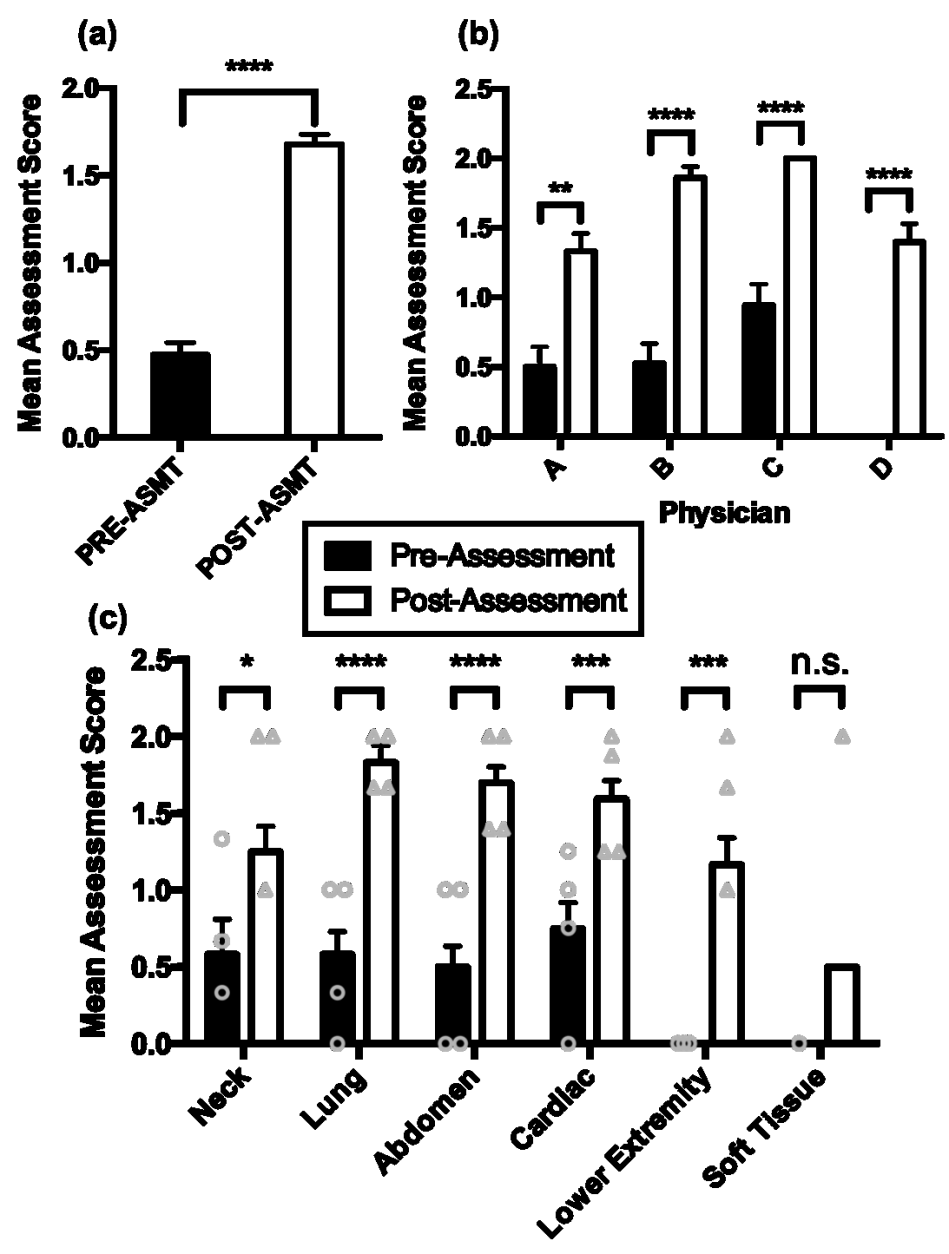
Mean assessment score of physicians pre- and post-training program with 95% confidence level. (a) The average POCUS competency score per assessment (trainees were given a 0, 1, or 2) pre- and post-training. Paired t-test, df=66, p < 0.0001. (b) The average POCUS competency score per assessment pre- and post-training for each physician that completed the entirety of the training program. Paired t-test, df=14, 17, 18, and 14 for Physicians A, B, C, and D, respectively, p = 0.0032 for Physician A, p < 0.0001 for Physicians B - D. (c) The average POCUS competency score per assessment pre- and post-training for each of the six medically relevant categories, as per the delineated categories in our assessment. Paired sample t-test, df=8, 11, 19, 15, and 8 for neck, lung, abdomen, cardiac, and lower extremity/DVT, respectively. p = 0.0207, p < 0.0001, p < 0.0001, p = 0.0002, and p < 0.0001, for neck, lung, abdomen, cardiac, lower extremity/DVT, respectively. Due to limited data in soft tissue category, no statistical testing could be conducted in this category. Vertical lines above each bar indicate one standard error of the mean on either side of the mean; absence of a bar indicates a mean of 0, and absence of a line above a bar indicates no reportable standard error. PRE-ASMT = Pre-assessment; POST-ASMT =Post-assessment.

The assessment score data for our sample of n = 4 physicians that completed the training program is shown in Fig. 1. The mean score across all assessments in the fundamental areas of POCUS are shown in Fig. 1(a); prior to our program, the average score per assessment was 0.47, which improved to 1.68 (p < 0.0001) after the training program. Furthermore, each physician showed substantial overall improvement in POCUS competency (Fig. 1(b)), independent of the initial competency pre-training (p < 0.004). Finally, there was an overall improvement in POCUS application for five of the six medically relevant assessment categories tested: neck, lung, abdomen, cardiac, and lower extremity imaging (Fig. 1(c)).

## Discussion

The LMEM model demonstrates a statistically significant trend of improvement in most categories of assessment. Given that our sample population consisted of Haitian physicians, the baseline assessment scores shown in Fig. 1(b) is expected, as the Haitian medical education covers many of the fundamentals of neck, lung, abdomen, and cardiac anatomy. In contrast, trainees exhibited no prior knowledge regarding image acquisition and interpretation with respect to lower extremities/DVTs and soft tissue ultrasonography, which are often considered to be more advanced ultrasound techniques. Improvements in assessment scores were observed in all categories of ultrasound image acquisition and interpretation regardless of previous experience. 

One limitation to this study was the trainee dropout rate. The dropout of eight out of twelve clinicians could have led to attrition bias in the assessment scores. Compared to other ultrasound training programs reported in the literature 19, 20, our dropout rate is expected. Follow-up with the clinicians who did not complete the program would be informative to understand the attitudinal, psychomotor-skill related, cultural, language, and/or clinical barriers to learning POCUS in a resource-limited setting. Despite this dropout rate, the longitudinal nature of this ultrasound training program resulted in highly skilled POCUS trainees, a few of which extended their clinical employment with the Alma Mater Hospital to continue their POCUS training of the remaining clinicians, including some that did not finish the training program described herein. These new POCUS-trained clinicians serve as evidence for the efficacy of the longitudinal model over the traditional short-term trips—by continually training local physicians, they are reducing the need for future volunteer trips.

Qualitatively, Physician C in Fig. 1(b) reported to have studied textbook ultrasound training material prior to the beginning of the program, while the other physicians reported no such self-studying 18. The data suggests that self-study, even without hands-on practice, could be beneficial for improved assessment scores both during the pre-study assessment and post-study assessment. Indeed, among all the study participants that completed this training program, Physician C performed the best in both the pre-training and post-training assessments conducted. Further randomized studies are needed with a larger number of study participants in order to better investigate if pre-program studying is beneficial to physician competency both during and following the program.

During this study, remote tele-ultrasound education was explored using the Reacts software integrated into the Philips Lumify ultrasound system. We found the Lumify with Reacts tele-ultrasound to be user-friendly and convenient even in this resource-limited setting. This suggests that Lumify with Reacts tele-ultrasound is a viable option for continued education and training even in resource-limited settings. In general, Lumify with the Reacts integration used in this study presents an exemplifying instance of the growing field of healthtech advancements, enabling long-term cost reductions and the ability to pursue sustainable LMEMs. This platform has the potential to empower local POCUS leaders to scale up training of other nearby communities with the support and expertise of international volunteer instructors, circumventing language/cultural barriers and high transportation costs, and reducing the target communities’ dependence on foreign involvement in the long run. This concept of self-sustainability is a particularly promising feature of the LMEM, as evidenced by the trainees training additional Haitian clinicians.

We envision the potential for many long-term benefits of pursuing LMEM as opposed to STMMs in terms of cost, sustainability, and self-reliability. As discussed previously, the primary limitation with most STMMs is that they are unable to build on previous work done with the communities of interest; every medical mission is essentially conducted independently of adjacent ones. Thus, while current STMMs can provide temporary relief with varying efficacy, they are not appropriately structured to provide medical assistance in the form of specialized medical education, technical training, and other forms of aid that cannot feasibly be accomplished within the mission’s short time frame. LMEM is a feasible solution to address this problem, and we have demonstrated its use in bringing POCUS education and technology to an under-resourced setting.

## Conclusions

The proposed LMEM model successfully demonstrates a pragmatic method of incorporating POCUS into an existing medical infrastructure in a resource-limited setting. Our POCUS training program significantly improved the clinician study participants’ ultrasound acquisition skills, and thereby patient care. By training local physicians through a longitudinal model and equipping them with state-of-the-art technology, we transcend many of the limitations suffered by short-term volunteering projects and promote self-sustainability. This eliminates the need for on-going volunteering trips thereby saving healthcare costs. Additional technological advancements, such as real-time tele-ultrasound training platforms and lower-cost ultrasound transducers will synergistically allow for more effective changes to community healthcare systems and improvements to differential diagnoses and patient treatments over the long term.

## Conflicts of Interest

The authors have no conflicts of interest to disclose.

## Ethics Approval

This study was deemed exempt and approved by the Legacy Emanuel Institutional Review Board (IRB) (Portland, OR) under exempt certification for research conducted in established or commonly accepted educational settings, involving normal educational practices. 

## Supplementary Material

Figure S1Featured Image